# Evaluation of ChatGPT as a Counselling Tool for Italian-Speaking MASLD Patients: Assessment of Accuracy, Completeness and Comprehensibility

**DOI:** 10.3390/jpm14060568

**Published:** 2024-05-26

**Authors:** Nicola Pugliese, Davide Polverini, Rosa Lombardi, Grazia Pennisi, Federico Ravaioli, Angelo Armandi, Elena Buzzetti, Andrea Dalbeni, Antonio Liguori, Alessandro Mantovani, Rosanna Villani, Ivan Gardini, Cesare Hassan, Luca Valenti, Luca Miele, Salvatore Petta, Giada Sebastiani, Alessio Aghemo, NAFLD Expert Chatbot Working Group

**Affiliations:** 1 Department of Biomedical Sciences, Humanitas University, Pieve Emanuele, 20072 Milan, Italy; nicola.pugliese@humanitas.it (N.P.); davide.polverini@humanitas.it (D.P.); cesare.hassan@hunimed.eu (C.H.); 2 Division of Internal Medicine and Hepatology, Department of Gastroenterology, IRCCS Humanitas Research Hospital, Rozzano, 20089 Milan, Italy; 3 Unit of Internal Medicine and Metabolic Disease, Fondazione IRCCS Ca’ Granda Ospedale Maggiore Policlinico of Milan, 20122 Milan, Italy; rosa.lombardi@unimi.it; 4 Department of Pathophysiology and Transplantation, Università degli Studi di Milano, 20122 Milan, Italy; luca.valenti@unimi.it; 5 Section of Gastroenterology and Hepatology, PROMISE, University of Palermo, 90127 Palermo, Italy; grazia.pennisi@unipa.it (G.P.); salvatore.petta@unipa.it (S.P.); 6 Department of Medical and Surgical Sciences (DIMEC), University of Bologna, 40138 Bologna, Italy; f.ravaioli@unibo.it; 7 Division of Internal Medicine, Hepatobiliary and Immunoallergic Diseases, IRCCS Azienda Ospedaliero Universitaria di Bologna, 40138 Bologna, Italy; 8 Division of Gastroenterology and Hepatology, Department of Medical Sciences, University of Turin, Corso Dogliotti 14, 10126 Turin, Italy; angelo.armandi@unito.it; 9 Metabolic Liver Disease Research Program, I. Department of Internal Medicine, University Medical Center of Mainz, 55131 Mainz, Germany; 10 Internal Medicine and Centre for Hemochromatosis and Hereditary Liver Diseases, ERN-EuroBloodNet Center for Iron Disorders, Azienda Ospedaliero-Universitaria di Modena-Policlinico, 41125 Modena, Italy; elena.buzzetti@unimore.it; 11 Department of Medical and Surgical Sciences, Università degli Studi di Modena e Reggio Emilia, 41125 Modena, Italy; 12 Division of General Medicine C, Department of Medicine, University and Azienda Ospedaliera Universitaria Integrata of Verona, University of Verona, 37134 Verona, Italy; andrea.dalbeni@univr.it; 13 Liver Unit, Department of Medicine, University and Azienda Ospedaliera Universitaria Integrata of Verona, University of Verona, 37134 Verona, Italy; 14 DiSMeC—Department of Scienze Mediche e Chirurgiche, Fondazione Policlinico Gemelli IRCCS, 00168 Rome, Italy; antonio.liguori@guest.policlinicogemelli.it (A.L.); luca.miele@policlinicogemelli.it (L.M.); 15 Section of Endocrinology, Diabetes and Metabolism, Department of Medicine, University and Azienda Ospedaliera Universitaria Integrata of Verona, Piazzale Stefani, 37126 Verona, Italy; alessandro.mantovani@univr.it; 16 C.U.R.E. (University Center for Liver Disease Research and Treatment), Liver Unit, Department of Medical and Surgical Sciences, University of Foggia, 71122 Foggia, Italy; rosanna.villani@unifg.it; 17 EpaC Onlus, Italian Liver Patient Association, 10141 Turin, Italy; gardini@epac.it; 18 Division of Gastroenterology and Digestive Endoscopy, Humanitas Research Hospital, IRCCS, Rozzano, 20089 Milan, Italy; 19 Precision Medicine Lab, Biological Resource Center, Department of Transfusion Medicine, Fondazione IRCCS Ca’ Granda Ospedale Maggiore Policlinico, 20122 Milan, Italy; 20 Department of Medicina e Chirurgia Traslazionale, Università Cattolica Del Sacro Cuore, 00168 Rome, Italy; 21 Division of Gastroenterology and Hepatology, McGill University Health Centre, Montreal, QC H4A 3J1, Canada; giada.sebastiani@mcgill.ca

**Keywords:** MASLD, artificial intelligence, counselling, diet, physical activity, steatosis, chatbot

## Abstract

Background: Artificial intelligence (AI)-based chatbots have shown promise in providing counseling to patients with metabolic dysfunction-associated steatotic liver disease (MASLD). While ChatGPT3.5 has demonstrated the ability to comprehensively answer MASLD-related questions in English, its accuracy remains suboptimal. Whether language influences these results is unclear. This study aims to assess ChatGPT’s performance as a counseling tool for Italian MASLD patients. Methods: Thirteen Italian experts rated the accuracy, completeness and comprehensibility of ChatGPT3.5 in answering 15 MASLD-related questions in Italian using a six-point accuracy, three-point completeness and three-point comprehensibility Likert’s scale. Results: Mean scores for accuracy, completeness and comprehensibility were 4.57 ± 0.42, 2.14 ± 0.31 and 2.91 ± 0.07, respectively. The physical activity domain achieved the highest mean scores for accuracy and completeness, whereas the specialist referral domain achieved the lowest. Overall, Fleiss’s coefficient of concordance for accuracy, completeness and comprehensibility across all 15 questions was 0.016, 0.075 and −0.010, respectively. Age and academic role of the evaluators did not influence the scores. The results were not significantly different from our previous study focusing on English. Conclusion: Language does not appear to affect ChatGPT’s ability to provide comprehensible and complete counseling to MASLD patients, but accuracy remains suboptimal in certain domains.

## 1. Introduction

Metabolic dysfunction-associated steatotic liver disease (MASLD) represents a significant global public health concern, anticipated to emerge as the foremost indication for liver transplantation in the coming years [1,2,3,4]. Its prevalence ranges between 20% and 40% in the general population, with a higher incidence observed among individuals struggling with obesity, type 2 diabetes (T2D), and/or dyslipidemia [1,2,3]. The spectrum of MASLD encompasses diverse pathological presentations ranging from simple hepatic steatosis (SLD) to metabolic dysfunction-associated steatohepatitis (MASH), characterized by inflammation, hepatocellular ballooning and fibrosis, predisposing to severe hepatic sequelae such as cirrhosis, end-stage liver disease and hepatocellular carcinoma (HCC) [4,5]. The increasing prevalence of T2D and obesity worldwide is expected to lead to a parallel increase in MASLD and MASH cases, further exacerbating the burden on healthcare systems worldwide [1].

Despite the recent Food and Drug Administration (FDA) approval of Resmetirom as the first prescribable drug for MASH and ongoing phase 3 trials investigating promising agents, lifestyle modification, particularly dietary changes and increased physical activity geared towards achieving weight reduction, remain pivotal in the management of MASLD [6,7,8,9,10,11]. Research has shown that weight loss, achievable through lifestyle modifications, significantly improves liver histology, diminishing inflammation, hepatocellular ballooning and fibrosis in patients with MASH [12,13].

Innovative strategies such as personalized counselling, behavioral interventions, and educational initiatives are essential to facilitate the adoption and perpetuation of lifestyle modifications among patients with MASLD. Given the inherent challenges in implementing lifestyle changes, comprehensive counselling assumes paramount importance to engendering patient motivation. Armed with comprehensive information about their condition, patients are empowered to cultivate an awareness that catalyzes habitual changes in diet and physical activity [14,15,16,17].

The growing interest in using artificial intelligence (AI) technologies in healthcare settings to deliver personalized support and patient education underscores the potential utility of AI-based chatbots. These conversational agents, which use natural language processing, have demonstrated efficacy in various healthcare settings, including mental health, chronic disease management and medication adherence [18,19,20]. By providing patients with round-the-clock support and information, AI-based chatbots complement existing healthcare resources, addressing patient queries and concerns regarding their condition. The OpenAI Foundation’s development of ChatGPT, an AI-based chatbot predicated on the third-generation Generative Pretrained Transformer-3.5 (GPT-3.5) architecture, has received widespread acclaim for its ability to handle various question-answer tasks [21,22]. 

A recent study conducted by our research group evaluated the potential of ChatGPT as a counselling tool for English-speaking patients with MASLD. While the initial findings were promising, they also highlighted discrepancies in the accuracy of ChatGPT-generated responses [23]. Presently, there is a paucity of research investigating the efficacy of chatbot-mediated counselling in non-English speaking MASLD patients, especially in the field of hepatology [24]. 

Against this backdrop, our study aims to assess the accuracy, completeness and comprehensibility of ChatGPT responses to basic questions posed in Italian by MASLD patients seeking insight into their condition. Specifically, we aim to determine whether language influences the quality of ChatGPT’s counselling by comparing its performance in answering MASLD-related questions in Italian to previous results in English. The insights gained from this study could have a significant impact on the use of AI-based tools in multilingual healthcare settings, providing tailored support to a wider range of patients and potentially improving the management and outcomes of MASLD worldwide.

## 2. Methods

### 2.1. Study Design

This study was conducted in October 2023 and was exempt from Institutional Review Board approval as it did not include patient-level data. 

The study used English-language questions from a previous investigation designed to evaluate the efficacy of ChatGPT as a counselling tool for patients with MASLD [23]. These questions were carefully translated into Italian to ensure linguistic accuracy and cultural relevance. The translation process involved a linguistic expert, fluent in both English and Italian, who meticulously adapted the questions to maintain their original intent and clarity in the target language (Table 1). 

In the original study, questions were formulated based on guideline-based preventive measures and interventional therapies, categorizing them into the following three domains: specialist referral, dietary composition and physical activity [9,10]. The specific questions included in this task are as follows:

(1) Specialist referral: questions 1, 14 and 15 addressed the need for specialist referral and the ability to detect improvement or worsening of MASLD.
(2) Dietary composition: Questions from 2 to 9 and 13 focused on dietary composition, weight loss, medication, coffee consumption, and alcohol consumption.
(3) Physical activity: questions 10, 11 and 12 asked about the type of physical activity recommended for MASLD patients.

The selection of the questions and domains was informed by established guidelines and best practices in MASLD management, ensuring that the assessment covered a comprehensive range of topics relevant to patient counselling. 

ChatGPT, an AI-based natural language processing model developed by OpenAI, was used as the counselling interface for this study. ChatGPT, a variant of the GPT-3.5 Large Language Model (LLM), has undergone extensive pre-training on a large dataset of online text up to 2021 [13]. This pre-training enables ChatGPT to generate responses that are linguistically coherent and contextually appropriate. In addition, ChatGPT has been continuously refined through human feedback, increasing its accuracy and reliability in generating relevant responses.

On 1 October 2023, two Milan-based researchers, Nicola Pugliese and Alessio Aghemo, entered the translated questions into ChatGPT using the March 14 version of the ‘New Chat’ feature. Each question was entered individually as an autonomous prompt to ensure independent and unbiased responses from the AI [25].

### 2.2. Evaluation of the AI-Generated Questions and Answers Section

The results of ChatGPT’s output were emailed to the thirteen Italian-speaking experts in MASLD (Supplementary Materials), including four senior experts who had previously evaluated ChatGPT’s performance in English (Luca Valenti, Luca Miele, Salvatore Petta, Giada Sebastiani), and nine junior experts specifically focused on metabolic liver diseases (Rosa Lombardi, Grazia Pennisi, Federico Ravaioli, Angelo Armandi, Elena Buzzetti, Rosanna Villani, Andrea Dalbeni, Alessandro Mantovani, Antonio Liguori) [23]. The experts involved in the evaluation come from different universities located in different regions of Italy (Piemonte, Lombardia, Veneto, Lazio, Emilia-Romagna, Puglia, Sicilia), thus ensuring a geographically diversified representation. The senior experts selected have an H-index of at least 40, while the junior experts have an H-index of at least 10. These H-index values were determined using Scopus at the time of the evaluation. This targeted selection aimed to include experts with a wide range of experience and expertise in the field of metabolic liver diseases, thus ensuring a comprehensive and accurate assessment of the responses generated by ChatGPT.

Additionally, the responses generated by ChatGPT were shared with an Italian-speaking non-physician (Ivan Gardini) with expertise in patient advocacy for liver disease.

Each expert was instructed to independently and blindly evaluate each AI-generated response using Likert scales to assess accuracy, completeness and comprehensiveness, drawing on their expertise and experience with MASLD [26]. The non-physician was specifically asked to rate the responses only for comprehensiveness using the same Likert scale.

For accuracy, a six-point Likert scale was employed, ranging from 1 (completely incorrect) to 6 (correct), allowing experts to gauge the degree of correctness in the responses.

Completeness was assessed using a three-point Likert scale, with rating ranging from 1 (incomplete, addresses some aspects of the question, but significant parts are missing or incomplete) to 3 (comprehensive, addresses all aspects of the question and provides additional information or context beyond what was expected), indicating the extent to which the responses addressed all aspects of the questions posed. 

Moreover, the comprehensibility of the text was evaluated using a separate three-point Likert scale, with ratings reflecting the ease of understating, ranging from 1 (difficult to understand) to 3 (easy to understand). 

For detailed Likert scale criteria, Supplementary Table S1 provides further clarification.

### 2.3. Data Collection and Statistical Analysis

The experts were instructed to rate each answer blindly.

The investigation findings underwent analysis utilizing descriptive statistics and concordance measures. Descriptive statistics were employed to compute the mean and standard deviation of the ratings for each question. Evaluations from the two groups of specialists (senior and young experts) underwent a t-test, with statistical significance determined at a threshold of *p* < 0.05. In addition, the senior experts’ ratings of ChatGPT-generated responses in Italian were compared with their ratings of English responses from a previous study by the NAFLD Expert Chatbot Working Group [23]. Concordance among experts was assessed using Fleiss’s Kappa, a non-parametric measure determining the degree of agreement among raters, considering both the magnitude and direction of differences. Interpretation of Kappa values followed the Landis and Koch criteria as follows: poor (<0.00), slight (0.00–0.20), fair (0.21–0.40), moderate (0.41–0.60), substantial (0.61–0.80), and almost perfect agreement (0.81–1.00).

## 3. Results

### 3.1. Accuracy

The survey results revealed that ChatGPT’s responses to 15 questions regarding MASLD were accurate, with an average score of 4.57 ± 0.42 on a Likert scale ranging from 1 to 6. Variations in scores were observed across different questions, with questions 9 (“Can I smoke if I have steatotic liver disease?”) and 10 (“How much exercise should I do if I have steatotic liver disease?”) achieving the highest scores, with mean scores of 5.07 ± 0.95 and 5.07 ± 0.86, respectively. Conversely, the lowest-scoring questions were questions 14 (“How do I understand if steatotic liver disease is worsening?”) and 15 (“How do I understand if steatotic liver disease is improving?”), averaging 3.84 ± 1.21 and 3.84 ± 1.14, respectively. 

**Figure 1:**
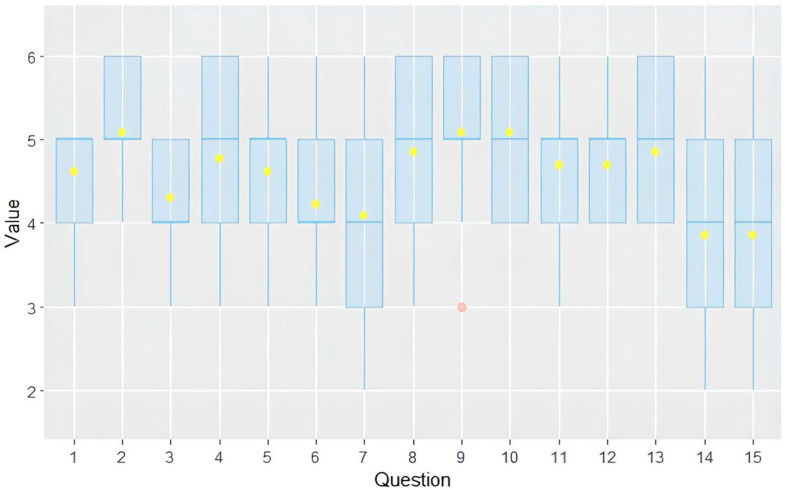
Box plot showing the distribution of accuracy scores. Graph shows interquartile range (box), median (horizontal line), mean (yellow dot) and outliers (whiskers).

In terms of domain-specific accuracy, the physical activity domain received the highest mean score of 4.82 ± 0.22, while the specialist referral domain received the lowest score with 4.1 ± 0.44 (see Figure 1).

No significant differences were observed in the evaluation of responses between senior and junior experts, with mean scores of 4.23 ± 0.6 and 4.72 ± 0.42, respectively (*p* = 0.11).

Similarly, there were no significant difference in the evaluation of Italian and English responses among senior experts, with mean scores of 4.23 ± 0.6 and 4.76 ± 0.48, respectively (*p* = 0.21). 

Agreement among experts was slight, with an overall Fleiss’s coefficient of 0.016.

### 3.2. Completeness 

The mean score for completeness was 2.14 ± 0.31 on a Likert scale ranging from 1 to 3. Among the individual questions, question 13 (“Which drugs should I take if I have steatotic liver disease?”) received the highest score of 2.53 ± 0.51, while question 15 (“How do I understand if steatotic liver disease is improving?”) received the lowest score of 1.46 ± 0.66. Notably, only five responses (questions 1, 2, 11, 13, and 19) received unanimous evaluation scores of 2 and 3. 

**Figure 2:**
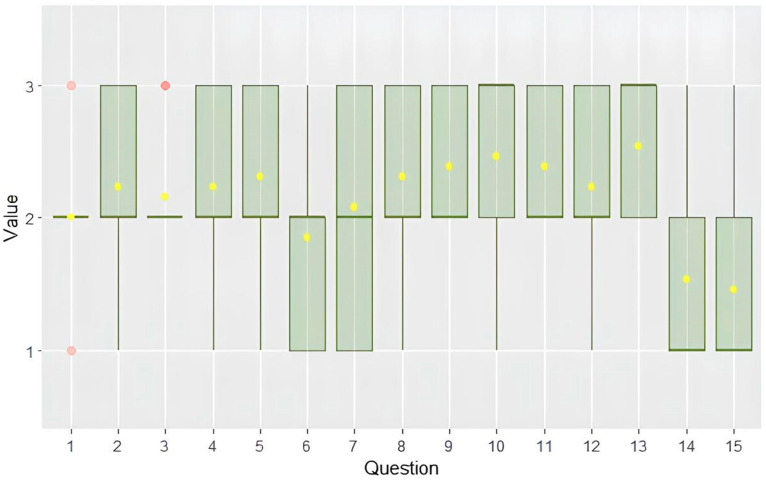
Box plot showing the distribution of completeness scores. Graph shows interquartile range (box), median (horizontal line), mean (yellow dot) and outliers (whiskers).

In terms of domain-specific completeness, the physical activity domain obtained the highest mean score of 2.35 ± 0.11, while the specialist referral domain had the lowest score of 1.66 ± 0.29 (see Figure 2).

Similar to the evaluation of accuracy, no significant differences were found in how senior experts evaluated the responses compared to the junior ones, with mean scores of 1.98 ± 0.36 and 2.21 ± 0.35, respectively (*p* = 0.3).

Additionally, there were no significant differences in the scoring of Italian and English responses among senior experts, with mean scores of 1.98 ± 0.36 and 1.79 ± 0.36, respectively (*p* = 0.48).

Agreement among experts was slight, with an overall Fleiss’s coefficient of 0.075.

### 3.3. Comprehensibility

The survey results indicate that ChatGPT’s responses to the 15 questions on MASLD received an average score of 2.91 ± 0.07 on the Likert scale ranging from 1 to 3. Notably, questions 1 (“I have been diagnosed with steatotic liver disease. Should I be referred to a liver specialist? Should I have additional blood tests or radiological examinations?”), 6 (“Which foods have a positive effect on steatotic liver disease?”), 7 (“Can I use herbal remedies if I have steatotic liver disease?”), and 8 (“Can I drink coffee if I have steatotic liver disease?”) received unanimous scores of 3, suggesting a high level of comprehensibility in these responses. However, agreement among experts was poor, with an overall Fleiss’s coefficient of −0.010. 

**Figure 3:**
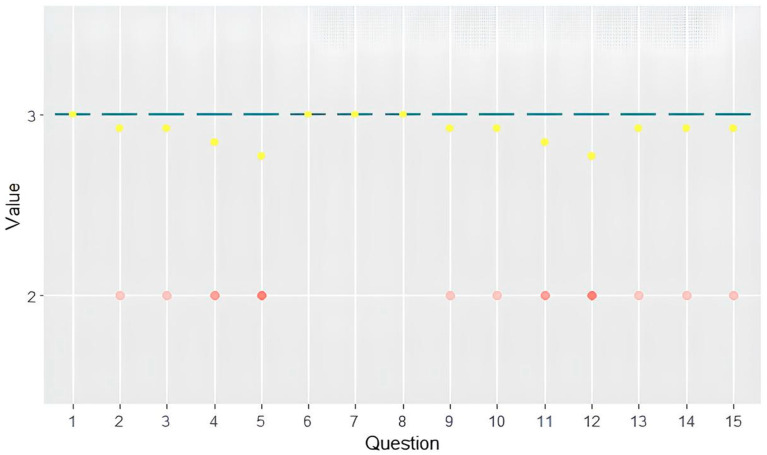
Box plot showing the distribution of comprehensibility scores. Graph shows interquartile range (box), median (horizontal line), mean (yellow dot) and outliers (whiskers).

Comprehensibility was also assessed by a non-physician who rated all questions as 3, indicating a consistent perception of high comprehensibility across all responses (see Figure 3).

Similarly to the evaluation of accuracy and completeness, no significant differences were observed in the evaluation of responses by senior experts compared to junior experts, nor in the assessment of Italian and English responses by senior experts.

## 4. Discussion and Conclusions

AI is anticipated to have a substantial impact on the medical field in the coming years and decades, affecting daily clinical practice [21,27,28]. Current research has primarily focused on developing AI-based tools to improve diagnostic accuracy and reduce inter- or intra-observer variability. However, the implementation of AI language model-based chatbots, like ChatGPT, has the potential to revolutionize the field by providing patients with immediate, free and on-demand counselling. 

MASLD is a significant global public health issue, projected to become the primary indication for liver transplantation in the coming decades. This is due to the increasing prevalence of metabolic syndrome and obesity, key risk factors for MASLD [1,2,3]. Lifestyle changes have proven beneficial, making MASLD an ideal context to test the accuracy, completeness, and comprehensibility of ChatGPT-generated responses to patients’ questions about managing their disease [9,10,12,13]. 

In a recent study, our group evaluated the performance of ChatGPT 3.5 in addressing MASLD-related questions. While the chatbot provided understandable responses, its accuracy could have been improved [11]. Notably, the study did not delve into the role and impact of the language used to interact with the chatbot. 

The current study aimed to assess the accuracy, completeness, and comprehensibility of ChatGPT 3.5 in answering 15 pre-determined MASLD-related questions in Italian. Thirteen Italian-speaking MASLD experts participated, ensuring a diverse and geographically representative sample. The results showed that ChatGPT’s ability to provide complete (mean score on a 3-point scale of 2.14) and understandable (mean score on a 3-point scale of 2.91) counseling to patients with MASLD remained consistent across different languages. However, the accuracy of ChatGPT responses remained suboptimal, with a mean score of 4.57 on a 6-point scale. Nonetheless, the responses were highly rated for comprehensibility, with five out of fifteen questions receiving the maximum grade from the experts. This was corroborated by the non-physician evaluator who also assigned the maximum grade to all responses. The results pertaining to completeness were also promising, albeit with only light agreement among experts in evaluating ChatGPT responses. 

Recent studies underscore accuracy as a critical area requiring improvement to prevent the dissemination of potentially harmful or erroneous information to patients [11,29]. For instance, in question 15, ‘How do I understand if steatotic liver disease is improving?’, six out of ten experts graded the answer ≤ 3, indicating misinformation in the chatbot-generated response. Interestingly, five of these six experts also found the response incorrect but easy to comprehend. Hence, a notable limitation of ChatGPT as a counselling tool is its propensity to generate inaccurate or hallucinated information [30]. While our study demonstrates high completeness on average, it is essential to acknowledge ChatGPT’s potential to yield incomplete or inaccurate responses, particularly in healthcare, where misinformation can have severe consequences. For example, the risk of ‘hallucinations’ raises concerns about the tool generating content that may not be grounded in evidence-based medical knowledge. Acting on health information without consulting a healthcare professional can have adverse health consequences. 

Furthermore, the influence of socio-cultural factors on ChatGPT responses warrants consideration. The tool’s ability to provide culturally sensitive advice depends on the patient’s socio-cultural context, and the effectiveness of the advice may vary based on cultural nuances, health literacy levels, and patient preferences. Therefore, while ChatGPT represents a valuable resource, its utilization must be approached with an understanding of patients’ cultural diversity [31].

This research is relevant as it is the first to evaluate the performance of ChatGPT as a counselling tool for Italian-speaking patients. While previous studies have explored the use of AI language models in English-speaking contexts, this study addresses the crucial need for such tools also in non-English speaking environments, particularly among Italian-speaking MASLD patients. Additionally, it benefits from the participation of experts from different age groups and academic positions in evaluating ChatGPT responses. The participation of a panel of highly qualified experts in the field of MASLD ensures the robustness of the evaluations and the applicability of the results to clinical practice. Moreover, the independent and blind evaluation of responses by experts helps to reduce the risk of bias and ensures the objectivity of the assessments. Lastly, the inclusion of a non-medical evaluator with specific expertise in patient advocacy for liver diseases offers a complementary perspective and enriches our understanding of the accessibility and usefulness of AI-based counselling resources for a wide range of users.

However, it is acknowledged that this study has several limitations. Initially, we enlisted a restricted group of experts to assess the answers based on subjective and self-reported ratings, using Likert scales. However, this method may not be optimal for evaluating medical texts, as it allows responses to be classified as ‘nearly all correct’. In a medical context, an answer that is ‘almost’ correct can still be significantly incorrect, with potential adverse consequences. Another potential limitation to consider is the availability of new and potentially improved versions of ChatGPT (e.g., ChatGPT 4). Nonetheless, it should be noted that, not being freely accessible, it may be difficult for patients to access. 

In conclusion, while our study presents promising results, the universal adoption of ChatGPT as a stand-alone counselling tool poses challenges. The identified limitations, particularly the potential for hallucinatory content and socio-cultural conditioning, underscore the need for continued refinement and validation of AI models in healthcare settings. For the future integration of ChatGPT or similar tools, collaboration between AI developers, healthcare professionals, and cultural experts is imperative in order to improve accuracy, cultural sensitivity, and overall reliability.

## Figures and Tables

**Table 1:** Questions posed to ChatGPT.

	Questions in English [11]	Questions in Italian
1	I have been diagnosed with steatotic liver disease. Should I be referred to a liver specialist? Should I have additional blood tests or radiological examinations?	Mi è stata diagnosticata la steatosi epatica. Devo rivolgermi ad un epatologo? Devo sottopormi ad esami del sangue o ad esami radiologici?
2	How much weight should I lose if I have steatotic liver disease?	Quanto peso devo perdere se ho la steatosi epatica?
3	Which diet should I follow if I have steatotic liver disease?	Quale dieta devo seguire se ho la steatosi epatica?
4	Which foods should I avoid if I have steatotic liver disease?	Quali cibi devo evitare se ho la steatosi epatica?
5	Can I drink alcohol if I have steatotic liver disease?	Posso bere alcolici se ho la steatosi epatica?
6	Which foods have a positive effect on steatotic liver disease?	Quali cibi hanno un effetto positivo sulla steatosi epatica?
7	Can I use herbal remedies if I have steatotic liver disease?	Posso usare rimedi erboristici per la mia steatosi epatica?
8	Can I drink coffee if I have steatotic liver disease?	Posso bere caffè se ho la steatosi epatica?
9	Can I smoke if I have steatotic liver disease?	Posso fumare se ho la steatosi epatica?
10	How much exercise should I do if I have steatotic liver disease?	Quanta attività fisica devo svolgere se ho la steatosi epatica?
11	Which type of exercise is better for steatotic liver disease?	Qual è la migliore tipologia di attività fisica per la steatosi epatica?
12	Should I do cardio or lift weights for steatotic liver disease?	Dovrei fare cardiofitness o sollevare pesi se ho la steatosi epatica?
13	Which drugs should I take if I have steatotic liver disease?	Quali farmaci devo assumere se ho la steatosi epatica?
14	How do I understand if steatotic liver disease is worsening?	In che modo mi rendo conto che la mia steatosi epatica è in peggioramento?
15	How do I understand if steatotic liver disease is improving?	In che modo mi rendo conto che la mia steatosi epatica è in miglioramento?

## Data Availability

The original contributions presented in the study are included in the article/Supplementary Materials; further inquiries can be directed to the corresponding author.

## Supplementary Materials

The following supporting information can be downloaded at: https://www.mdpi.com/article/10.3390/jpm14060568/s1, Table S1: Likert scales.
