# Supplementary material for: Evaluation of ChatGPT as a Counselling Tool for Italian-Speaking MASLD Patients: Assessment of Accuracy, Completeness and Comprehensibility
Source: J Pers Med. 2024 May 26;14(6):568. doi: 10.3390/jpm14060568 (PMC11204905; doi:10.3390/jpm14060568)
Supplement: Supplementary file 1 [file jpm-14-00568-s001.zip › jpm-3015361-supplementary.pdf]

Supplementary Table S1 Likert scales

	ACCURACY RATING BY A SIX-POINT LIKERT SCALE
**1**	completely incorrect
**2**	more incorrect than correct
**3**	approximately equal correct and incorrect
**4**	more correct than incorrect
**5**	nearly all correct
**6**	correct

	COMPLETENESS RATING BY A THREE-POINT LIKERT SCALE
**1**	incomplete, addresses some aspects of the question, but significant parts are missing or incomplete
**2**	adequate, addresses all aspects of the question and provides the minimum amount of information required to be considered complete
**3**	comprehensive, addresses all aspects of the question and provides additional information or context beyond what was expected

	COMPREHENSIBILITY RATING BY A THREE-POINT LIKERT SCALE
**1**	difficult to understand
**2**	partly difficult to understand
**3**	easy to understand

Supplementary material: QUESTIONS POSED TO CHATGPT (ITALIAN)

1. **INPUT** -> Mi è stata diagnosticata la steatosi epatica. Devo rivolgermi ad un epatologo? Devo sottopormi ad esami del sangue o ad esami radiologici?

OUTPUT:

Sono lieto di darti alcune informazioni sulla steatosi epatica, ma ricorda che non sono un medico e le mie risposte sono solo a scopo informativo. È importante consultare un medico o un epatologo per ottenere consigli e trattamenti specifici basati sulla tua situazione individuale.

La steatosi epatica, comunemente nota come "fegato grasso", è una condizione in cui il grasso si accumula nelle cellule del fegato. Esistono due tipi principali di steatosi epatica:

1. Steatosi epatica non alcolica (NAFLD): Questa è la forma più comune di steatosi epatica e non è causata dall'abuso di alcol. Può essere associata a fattori come l'obesità, il diabete, l'ipertensione e l'iperlipidemia.
2. Steatosi epatica alcolica (ALD): Questa forma è causata dall'abuso cronico di alcol.

La gestione della steatosi epatica dipenderà dalla sua causa e dalla gravità della condizione. Tuttavia, in generale, ecco alcune misure comuni che possono essere raccomandate:

1. Modifiche dello stile di vita: La perdita di peso, l'esercizio fisico regolare e una dieta equilibrata possono aiutare a ridurre il grasso nel fegato. Queste misure possono essere particolarmente importanti se hai NAFLD.
2. Limitare l'assunzione di alcol: Se hai ALD, sarà importante ridurre o eliminare l'assunzione di alcol.
3. Monitoraggio medico: Sarà importante fare regolari visite mediche per controllare la tua condizione e valutare il tuo progresso.
4. Esami del sangue e imaging: Il tuo medico potrebbe consigliarti di sottoporsi a esami del sangue per monitorare la funzionalità epatica e potrebbe richiedere esami di imaging come l'ecografia, la tomografia computerizzata (TC) o la risonanza magnetica (RM) per valutare il grado di accumulo di grasso nel fegato.
5. Consulto con un epatologo: Se la tua condizione è più grave o complicata, potrebbe essere necessario consultare un epatologo, un medico specializzato nelle malattie del fegato. L'epatologo sarà in grado di fornirti una consulenza più approfondita e pianificare un trattamento specifico, se necessario.

Ricorda che la gestione della steatosi epatica può variare da persona a persona, quindi è importante seguire attentamente le indicazioni del tuo medico per ottenere il miglior risultato possibile. Non esitare a rivolgerti al tuo medico di fiducia per discutere ulteriormente della tua situazione e delle opzioni di trattamento

2. **INPUT ->** Quanto peso devo perdere se ho la steatosi epatica?

OUTPUT:

La quantità di peso che dovresti perdere se hai la steatosi epatica dipenderà dalla tua situazione individuale, dalla gravità della condizione e da altri fattori, come il tuo peso corporeo attuale, l'età, il sesso e le condizioni di salute generali. Non esiste una risposta universale in termini di quanti chili dovresti perdere.

In generale, la perdita di peso può essere utile nella gestione della steatosi epatica non alcolica (NAFLD), specialmente se sei in sovrappeso o obeso. Anche una modesta riduzione del peso corporeo può portare a miglioramenti significativi nella salute del fegato. Tuttavia, è importante sottolineare che una perdita di peso sana e sostenibile dovrebbe essere gradualmente raggiunta e dovrebbe essere supervisionata da un professionista medico o un dietologo registrato.

La quantità di peso che dovresti mirare a perdere può variare, ma in molti casi, una perdita del 5-10% del peso corporeo totale può avere un impatto positivo sulla salute del fegato. Ad esempio, se il tuo peso corporeo attuale è di 90 kg, una perdita di peso del 5-10% sarebbe equivalente a 4,5-9 kg. Tuttavia, è importante discutere con il tuo medico o un professionista della salute qualificato per stabilire obiettivi di perdita di peso specifici basati sulla tua situazione e sulle tue esigenze.

La chiave per una perdita di peso efficace e duratura è adottare uno stile di vita sano che includa una dieta equilibrata, esercizio fisico regolare e cambiamenti sostenibili nelle abitudini alimentari. L'obiettivo dovrebbe essere quello di migliorare la salute generale e non solo la condizione del fegato. Un medico o un dietologo può aiutarti a sviluppare un piano personalizzato che tenga conto delle tue esigenze e obiettivi specifici.

3. **INPUT ->** Quale dieta devo seguire se ho la steatosi epatica?
OUPUT:

La dieta per la steatosi epatica dovrebbe essere sviluppata in collaborazione con un professionista medico o un dietologo registrato, poiché la dieta esatta può variare in base alla gravità della tua condizione, alle tue esigenze individuali e ad altri fattori di salute. Tuttavia, posso darti alcune linee guida generali per una dieta che può aiutare a gestire la steatosi epatica:

1. Riduci l'apporto calorico: Se sei in sovrappeso o obeso, la perdita di peso può essere un obiettivo importante. Ridurre gradualmente l'apporto calorico può aiutare a perdere peso in modo sano e sostenibile. Consulta un professionista della salute per stabilire il tuo fabbisogno calorico giornaliero ottimale.
2. Limita l'assunzione di grassi saturi e trans: Riduci l'apporto di alimenti ad alto contenuto di grassi saturi e grassi trans, come carni grasse, latticini interi, cibi fritti e prodotti da forno con grassi idrogenati. Invece, opta per grassi più sani come quelli presenti in avocado, noci, semi e oli vegetali come l'olio d'oliva.
3. Scegli proteine magre: Le proteine magre, come il pollo senza pelle, il pesce, il tofu e le leguminose, possono essere una scelta migliore rispetto alle carni rosse grasse. Riduci l'apporto di carne rossa e lavorata.
4. Aumenta l'apporto di fibre: Le fibre possono aiutare a controllare il peso e a migliorare la salute del fegato. Scegli alimenti ricchi di fibre come frutta, verdura, cereali integrali, legumi e noci.
5. Limita l'assunzione di zuccheri aggiunti: Riduci l'apporto di zuccheri aggiunti, come quelli presenti nelle bevande zuccherate, dolci, biscotti e cibi processati.
6. Monitora l'assunzione di carboidrati: Alcune persone con steatosi epatica possono beneficiare da una moderata limitazione dell'apporto di carboidrati, specialmente quelli ad alto indice glicemico. Un professionista della salute può aiutarti a pianificare un consumo di carboidrati adatto alla tua situazione.
7. Evita l'alcol: Se hai la steatosi epatica alcolica (ALD), è essenziale eliminare o ridurre drasticamente il consumo di alcol.
8. Bevi acqua: Mantieni l'idratazione bevendo abbondante acqua durante il giorno.
9. Suddividi i pasti: Mangiare piccoli pasti frequenti può aiutare a mantenere stabili i livelli di zucchero nel sangue e la sensazione di sazietà.

Ricorda che questi sono solo consigli generali e che la dieta dovrebbe essere adattata alle tue esigenze specifiche. È importante consultare un professionista medico o un dietologo registrato che possa valutare la tua situazione individuale e sviluppare un piano alimentare su misura per te. Inoltre, il monitoraggio regolare della tua condizione da parte di un medico è essenziale per assicurarti che stai ottenendo i risultati desiderati e per apportare eventuali aggiustamenti al tuo regime dietetico

4. **INPUT** -> Quali cibi devo evitare se ho la steatosi epatica?
OUTPUT:

Se hai la steatosi epatica, dovresti evitare o limitare l'assunzione di alcuni cibi e bevande che possono peggiorare la condizione del tuo fegato grasso. Ecco alcuni cibi e bevande che dovresti considerare di evitare o consumare con molta moderazione:

1. **Alcol**: Se hai la steatosi epatica alcolica (ALD), è fondamentale evitare completamente l'alcol. L'alcol può danneggiare ulteriormente il fegato e peggiorare la condizione.
2. **Alimenti ad alto contenuto di grassi saturi**: Riduci o elimina il consumo di cibi ricchi di grassi saturi, come carne grassa, burro, formaggi pieni di grassi e prodotti da forno contenenti grassi saturi idrogenati.
3. **Zuccheri aggiunti**: Limita l'assunzione di zuccheri aggiunti, come quelli presenti nelle bevande zuccherate, dolci, biscotti, cereali da colazione zuccherati e altri prodotti dolci.
4. **Cibi fritti**: Evita cibi fritti o cotti in grassi saturi o oli ad alto contenuto di grassi saturi.

5. **Cibi altamente processati**: Riduci l'apporto di cibi altamente processati, come snack confezionati, fast food e cibi pronti surgelati, poiché spesso contengono grassi saturi, zuccheri aggiunti e additivi che potrebbero non essere benefici per il fegato.
6. **Carne rossa e lavorata**: Limita il consumo di carne rossa grassa e di carni lavorate come salsicce, pancetta e hot dog.
7. **Bevande energetiche**: Evita le bevande energetiche e le bevande gassate zuccherate, poiché sono spesso ricche di zuccheri aggiunti e possono contribuire al sovrappeso.
8. **Sali e cibi ricchi di sodio**: Riduci il consumo di sale e di cibi ad alto contenuto di sodio, poiché l'alto consumo di sodio può portare a problemi di salute, inclusa l'ipertensione.
9. **Cibi con elevato indice glicemico**: Alcuni studi suggeriscono che una dieta con un alto indice glicemico può influenzare negativamente la steatosi epatica. Cerca di limitare i cibi con elevato indice glicemico, come il pane bianco, il riso bianco e i cereali raffinati.
10. **Bibite alcoliche senza alcool**: Alcune bevande senza alcol possono contenere zuccheri aggiunti o dolcificanti artificiali che potrebbero non essere benefici. Leggi le etichette degli ingredienti.

In generale, è consigliabile seguire una dieta equilibrata, ricca di frutta, verdura, cereali integrali, proteine magre e grassi sani come quelli presenti in avocado, noci e oli vegetali. Un dietologo o un professionista della salute può aiutarti a sviluppare un piano alimentare specifico per le tue esigenze individuali e per la gestione della steatosi epatica.

5. INPUT -> Posso bere alcolici se ho la steatosi epatica?

OUTPUT:

Se ti è stata diagnosticata la steatosi epatica, è fondamentale prendere decisioni informate sulla tua dieta e sul consumo di alcolici. In generale, l'alcol dovrebbe essere evitato o consumato con molta moderazione se hai la steatosi epatica, indipendentemente dalla sua forma (alcolica o non alcolica). Ecco alcune importanti considerazioni:

1. **Steatosi Epatica Alcolica (ALD)**: Se hai la forma alcolica della steatosi epatica, è estremamente consigliabile evitare completamente l'alcol. L'assunzione di alcol può peggiorare la condizione del tuo fegato e contribuire allo sviluppo di patologie epatiche più gravi come l'epatite alcolica e la cirrosi. La sospensione totale dell'alcol è il trattamento primario per l'ALD.
2. **Steatosi Epatica Non Alcolica (NAFLD)**: Anche se la forma non alcolica della steatosi epatica (NAFLD) non è causata dall'abuso di alcol, è comunque importante limitare o eliminare il consumo di alcol. L'alcol può mettere ulteriore stress sul tuo fegato e può rendere più difficile la gestione dell'NAFLD. Inoltre, spesso l'NAFLD è associata a fattori di rischio come obesità, diabete e sindrome metabolica, e l'alcol può influenzare negativamente questi fattori.
3. **Consulto Medico**: La tua decisione di consumare alcol dovrebbe essere discussa in dettaglio con un professionista medico, come un epatologo o un gastroenterologo. Il medico può valutare la gravità della tua condizione, i fattori di rischio e darti consigli personalizzati sulla base della tua situazione specifica. In molti casi, i medici raccomandano di evitare completamente l'alcol per proteggere il fegato.
4. **Monitoraggio Costante**: Indipendentemente dalla tua decisione sull'alcol, è importante sottoporsi a regolari visite mediche di monitoraggio per valutare la salute del tuo fegato e apportare eventuali modifiche al trattamento o allo stile di vita in base alla tua risposta alla terapia.

In sintesi, evitare completamente l'alcol è spesso la scelta più sicura per proteggere la salute del fegato se hai la steatosi epatica. Tuttavia, la decisione finale dovrebbe essere presa in consulto con il tuo medico, che considererà tutti i fattori pertinenti alla tua situazione clinica

6. INPUT -> Quali cibi hanno un effetto positivo sulla steatosi epatica?

OUTPUT:

Una dieta equilibrata e mirata può avere un effetto positivo sulla steatosi epatica, contribuendo a migliorare la salute del fegato. Ecco alcuni cibi che possono essere benefici per la steatosi epatica:

1. **Frutta e verdura**: Sono ricche di antiossidanti, vitamine, e fibre, che possono contribuire a ridurre l'infiammazione e migliorare la salute del fegato. Scegli una varietà di frutta e verdura colorate per massimizzare i benefici.
2. **Cereali integrali**: Alimenti come il pane integrale, il riso integrale, l'avena e la quinoa sono fonti di carboidrati complessi e fibre che possono aiutare a controllare i livelli di zucchero nel sangue e favorire la perdita di peso.
3. **Proteine magre**: Carne magra, pesce, pollame senza pelle, tofu e legumi sono buone fonti di proteine magre che possono essere incluse in una dieta per la steatosi epatica.
4. **Noci e semi**: Sono ricchi di grassi sani, tra cui acidi grassi omega-3, che possono avere un effetto positivo sulla salute del fegato. Tuttavia, le noci sono anche caloriche, quindi è importante consumarle con moderazione.
5. **Oli vegetali sani**: L'olio d'oliva extra vergine e l'olio di semi di lino sono esempi di oli vegetali sani che possono essere utilizzati per condire cibi e cucinare.
6. **Pesce grasso**: Il pesce grasso come il salmone, il tonno e le sardine è una buona fonte di acidi grassi omega-3, che possono avere proprietà anti-infiammatorie e contribuire alla salute del fegato.
7. **Tè verde**: Il tè verde è ricco di antiossidanti e può contribuire a ridurre l'infiammazione nel fegato.
8. **Latte e latticini a basso contenuto di grassi**: Il latte scremato, lo yogurt magro e il formaggio a basso contenuto di grassi possono essere scelte migliori rispetto ai loro equivalenti ricchi di grassi.
9. **Cibi ricchi di fibre**: I cibi ricchi di fibre, come i legumi, l'avena e le verdure a foglia verde, possono aiutare a regolare il transito intestinale e a migliorare il metabolismo dei grassi.
10. **Aceto di mele**: Alcuni studi suggeriscono che l'aceto di mele può avere effetti positivi sulla gestione del peso e sulla riduzione del grasso nel fegato.

Ricorda che la chiave per una dieta efficace per la steatosi epatica è la moderazione e la varietà. Inoltre, è importante consultare un professionista medico o un dietologo registrato per sviluppare un piano alimentare personalizzato in base alla tua situazione individuale e ai tuoi obiettivi di salute.

7. **INPUT ->** Posso usare rimedi erboristici per la mia steatosi epatica?
OUTPUT:

L'uso di rimedi erboristici per la steatosi epatica (fegato grasso) dovrebbe essere fatto con cautela e sotto la supervisione di un professionista medico. Mentre alcune erbe possono avere benefici per la salute epatica, è importante considerare diversi fattori:

1. **Consulto Medico**: Prima di iniziare qualsiasi trattamento a base di erbe per la steatosi epatica, è fondamentale consultare il tuo medico o un epatologo. Questo è importante perché è necessario confermare la diagnosi e determinare la causa sottostante della steatosi epatica. Inoltre, il tuo medico può valutare le potenziali interazioni tra gli integratori erboristici e altri farmaci che potresti essere in grado di assumere per altre condizioni mediche.
2. **Qualità e Sicurezza**: Assicurati di utilizzare prodotti erboristici di alta qualità da fonti affidabili. La sicurezza e la purezza dei prodotti erboristici possono variare notevolmente, quindi è importante fare acquisti da produttori o rivenditori di fiducia.
3. **Interazioni Farmacologiche**: Alcuni rimedi erboristici possono interagire con farmaci o altri trattamenti medici che stai seguendo. Informa il tuo medico o il professionista della salute sui rimedi erboristici che intendi utilizzare per evitare potenziali interazioni pericolose.
4. **Effetti Collaterali**: Anche gli integratori erboristici possono avere effetti collaterali o reazioni allergiche. Monitora attentamente la tua risposta ai rimedi erboristici e interrompi l'uso se noti effetti indesiderati.
5. **Evidenze Scientifiche Limitate**: La ricerca scientifica sugli effetti specifici degli integratori erboristici sulla steatosi epatica è spesso limitata o inconcludente. Molti rimedi erboristici non sono stati sottoposti a rigorose prove cliniche per valutarne l'efficacia.
6. **Approccio Integrato**: L'uso di rimedi erboristici dovrebbe essere parte integrante di un piano di gestione globale della steatosi epatica, che può includere anche modifiche dello stile di vita come una dieta equilibrata, l'esercizio fisico e la gestione del peso.

Alcune erbe comunemente associate alla salute del fegato includono il cardo mariano, il tarassaco, la curcuma e il trifoglio rosso. Tuttavia, è importante sottolineare che il trattamento della steatosi epatica dovrebbe essere basato su evidenze cliniche solide e supervisionato da un professionista medico.

In sintesi, se desideri utilizzare rimedi erboristici per la steatosi epatica, discutine sempre con il tuo medico o un epatologo e segui le loro raccomandazioni. La supervisione medica è essenziale per garantire che il trattamento sia sicuro ed efficace e che sia adeguato alla tua situazione individuale

8. **INPUT ->** Posso bere caffè se ho la steatosi epatica?
OUTPUT:

Il consumo moderato di caffè è generalmente considerato sicuro per le persone con la steatosi epatica (fegato grasso), sia che si tratti di steatosi epatica non alcolica (NAFLD) che di steatosi epatica alcolica (ALD). In realtà, alcune ricerche suggeriscono che il caffè potrebbe avere benefici per la salute epatica. Tuttavia, è importante fare alcune considerazioni:

1. **Moderazione**: Come per molte cose nella dieta, la chiave è la moderazione. Consumare caffè in quantità eccessive potrebbe non essere salutare per il fegato o per la salute generale. Di solito, una o due tazze di caffè al giorno sono considerate una quantità moderata.
2. **Tolleranza Individuale**: La tolleranza al caffè può variare da persona a persona. Alcune persone possono essere più sensibili agli effetti stimolanti della caffeina, quindi è importante ascoltare il proprio corpo e regolare il consumo di caffè in base alle proprie reazioni.
3. **Benefici Potenziali**: Alcuni studi hanno suggerito che il caffè potrebbe avere effetti benefici sulla salute del fegato. In particolare, si è notato che il caffè può contribuire a ridurre il rischio di sviluppare l'NAFLD e potrebbe avere un impatto positivo sulla progressione della malattia. Tuttavia, gli effetti possono variare da persona a persona.
4. **Attenzione agli Additivi**: Quando si beve caffè, è importante considerare gli additivi come lo zucchero e la crema. L'aggiunta di grandi quantità di zucchero e grassi saturi può avere effetti negativi sulla salute generale, compresa la salute del fegato.
5. **Consulto Medico**: Se hai preoccupazioni specifiche sulla tua salute epatica o se stai seguendo un trattamento medico per la steatosi epatica, è sempre una buona idea consultare il tuo medico o un epatologo per ottenere indicazioni personalizzate sul consumo di caffè.

In sintesi, il caffè può essere consumato con moderazione come parte di una dieta equilibrata, e potrebbe addirittura avere benefici per la salute del fegato grasso. Tuttavia, è importante essere consapevoli dei propri limiti e di consultare un professionista della salute se si hanno domande o preoccupazioni specifiche sulla relazione tra il caffè e la steatosi epatica.

9. **INPUT ->** Posso fumare se ho la steatosi epatica?
OUTPUT:

Fumare è dannoso per la salute in generale e può avere effetti negativi sul fegato, compreso il fegato grasso (steatosi epatica). Ecco alcune considerazioni importanti:

1. **Effetti Negativi del Fumo sul Fegato**: Il fumo di sigaretta è associato a una serie di effetti negativi sul fegato, tra cui un aumento del rischio di malattie del fegato, compresa la progressione della steatosi epatica verso forme più gravi come l'epatite alcolica o la cirrosi. Il fumo può anche contribuire a un'infiammazione cronica nel fegato.
2. **Interazioni con Altre Condizioni**: Se hai la steatosi epatica, è probabile che tu abbia anche altri fattori di rischio come l'obesità, il diabete o l'ipertensione. Fumare può peggiorare queste condizioni, rendendo più difficile il controllo della steatosi epatica e aumentando il rischio di complicazioni.
3. **Effetti sul Metabolismo dei Grassi**: Il fumo di sigaretta può alterare il metabolismo dei grassi nel fegato, contribuendo al deposito di grasso. Questo è particolarmente rilevante in caso di steatosi epatica, dove la gestione dei grassi nel fegato è già compromessa.
4. **Smettere di Fumare**: La scelta migliore per la tua salute generale e per la gestione della steatosi epatica sarebbe quella di smettere completamente di fumare. L'abbandono del fumo può migliorare la tua salute epatica e ridurre il rischio di gravi complicazioni epatiche nel lungo termine.
5. **Supporto per Smettere di Fumare**: Se desideri smettere di fumare, cerca il supporto di un professionista medico o di un programma di cessazione tabagica. Ci sono molte risorse disponibili per aiutarti a smettere di fumare in modo efficace.

In sintesi, se hai la steatosi epatica, smettere di fumare è una scelta importante per migliorare la salute del tuo fegato e del tuo corpo nel suo complesso. Consulta il tuo medico per ottenere supporto nella tua decisione di smettere di fumare e per ricevere consigli specifici sulla gestione della tua steatosi epatica.

10. INPUT -> Quanta attività fisica devo svolgere se ho la steatosi epatica?
OUTPUT:

L'attività fisica è un componente importante nella gestione della steatosi epatica (fegato grasso), in particolare se si tratta di steatosi epatica non alcolica (NAFLD). L'esercizio fisico può contribuire a migliorare la salute generale, a ridurre il peso corporeo, a migliorare la sensibilità all'insulina e a promuovere la salute del fegato. Tuttavia, il tipo e la quantità di attività fisica possono variare in base alle tue condizioni individuali e al livello di forma fisica.

Ecco alcune linee guida generali sulla quantità e il tipo di attività fisica da considerare:

1. **Consulenza Medica**: Prima di iniziare qualsiasi programma di esercizio, è importante consultare il tuo medico o un professionista della salute per assicurarti che sia sicuro per te, specialmente se hai condizioni mediche preesistenti o se stai seguendo trattamenti specifici per la steatosi epatica.
2. **Attività Aerobica**: Gli esercizi aerobici come camminare, nuotare, fare jogging, andare in bicicletta o ballare possono essere molto benefici per migliorare la salute del fegato e per la perdita di peso. Si consiglia di mirare a 150-300 minuti di attività aerobica moderata o 75-150 minuti di attività intensa ogni settimana, suddivisi su almeno 3-5 giorni.
3. **Esercizi di Forza**: Gli esercizi di forza o resistenza, come il sollevamento pesi o l'utilizzo di macchine per la resistenza, possono aiutare a sviluppare massa muscolare e a migliorare il metabolismo. Si consiglia di includere almeno due giorni alla settimana di esercizi di forza che coinvolgano tutti i principali gruppi muscolari.
4. **Flessibilità e Bilanciamento**: Esercizi di stretching e attività come lo yoga o il tai chi possono contribuire a migliorare la flessibilità, il bilanciamento e la coordinazione.
5. **Gradualità**: Se sei inattivo o hai una forma fisica limitata, inizia gradualmente e aumenta l'intensità e la durata dell'attività fisica man mano che ti senti più a tuo agio. L'obiettivo è quello di creare un programma di esercizio sostenibile nel tempo.
6. **Varietà**: Scegli una varietà di attività che ti piacciano per rendere l'esercizio più piacevole e sostenibile nel lungo termine.
7. **Persistenza**: La coerenza è fondamentale. Cerca di mantenere un programma di esercizio regolare nel tempo.
8. **Idratazione**: Assicurati di rimanere ben idratato durante e dopo l'attività fisica.
9. **Controllo Medico Periodico**: Continua a monitorare la tua condizione con il tuo medico e chiedi il suo parere su come adattare il tuo programma di esercizio in base alla tua risposta e ai tuoi obiettivi di salute.

In generale, l'esercizio fisico è un componente importante per migliorare la salute del fegato grasso, ma è essenziale essere consapevoli delle tue limitazioni e di consultare un professionista della salute per sviluppare un piano di esercizio personalizzato.

11. INPUT -> Qual è la migliore tipologia di attività fisica per la steatosi epatica?
OUTPUT:

La migliore tipologia di attività fisica per la steatosi epatica (fegato grasso) è una combinazione di esercizi aerobici (cardio), esercizi di resistenza (forza) e attività che migliorano la flessibilità e il bilanciamento. Un programma completo di attività fisica può apportare diversi benefici alla salute del fegato e alla gestione della steatosi epatica. Ecco come ogni tipo di attività può essere benefico:

1. Esercizi Aerobici (Cardio):

- Camminata: La camminata è un'opzione accessibile e può essere adatta a quasi tutti.
- Nuoto: È una forma di esercizio a basso impatto che coinvolge molti muscoli.
- Ciclismo: Ottimo per il cuore e per bruciare calorie.
- Corsa: Può essere più intensa ma efficace per migliorare la salute cardiovascolare.
- Danza: Una modalità divertente di esercizio aerobico.

2. Esercizi di Resistenza (Forza):

- Sollevamento Pesi: Puoi utilizzare pesi liberi o macchine per la resistenza.
- Esercizi con il Peso del Corpo: Come push-up, squat e affondi.
- Yoga o Pilates: Questi possono includere esercizi di forza per il core e il corpo in generale.

3. Esercizi per la Flessibilità e il Bilanciamento:

- Yoga: Favorisce la flessibilità e il rilassamento.
- Tai Chi: Contribuisce al miglioramento del bilanciamento e della coordinazione.
- Stretching: Aiuta a mantenere la flessibilità muscolare e a prevenire lesioni.

L'obiettivo è sviluppare un programma di attività fisica che combini queste diverse modalità, in modo da ottenere una gamma completa di benefici per la salute. Ecco alcune linee guida generali:

- Frequenza: Cerca di esercitarti almeno 3-5 giorni alla settimana.
- Durata: L'obiettivo è di almeno 150-300 minuti di esercizio aerobico moderato o 75-150 minuti di esercizio intenso ogni settimana, suddivisi su almeno 3-5 giorni. Includi anche almeno due giorni alla settimana di esercizi di resistenza che coinvolgono tutti i principali gruppi muscolari.
- Gradualità: Se sei inattivo o hai una forma fisica limitata, inizia gradualmente e aumenta l'intensità e la durata man mano che ti senti più a tuo agio.
- Consulto Medico: Consulta il tuo medico o un professionista della salute prima di iniziare un nuovo programma di esercizi, specialmente se hai condizioni mediche preesistenti o stai seguendo trattamenti specifici per la steatosi epatica.

In sintesi, la combinazione di esercizi aerobici, esercizi di resistenza e attività per la flessibilità e il bilanciamento costituisce il miglior approccio per migliorare la salute del fegato e gestire la steatosi epatica. Un professionista della salute può aiutarti a sviluppare un piano di esercizio personalizzato in base alle tue esigenze e alla tua situazione individuale.

12. INPUT -> Dovrei fare cardiofitness o sollevare pesi se ho la steatosi epatica?
OUTPUT:

Se hai la steatosi epatica (fegato grasso), è consigliabile combinare sia l'attività cardiofitness (aerobica) che l'allenamento con i pesi (resistenza) nel tuo programma di esercizio. Entrambi i tipi di attività fisica possono essere benefici per la gestione della steatosi epatica, ma svolgono ruoli leggermente diversi. Ecco perché è utile includere entrambi:

1. Cardiofitness (Esercizi Aerobici):

- Benefici: Gli esercizi aerobici, come camminare, nuotare, andare in bicicletta o correre, aiutano a bruciare calorie, migliorare la capacità cardiovascolare, ridurre il peso corporeo e aumentare la sensibilità all'insulina. Possono anche contribuire a ridurre l'accumulo di grasso nel fegato.
- Consigliato: Cerca di impegnarti in almeno 150-300 minuti di esercizi aerobici moderati o 75-150 minuti di esercizi intensi ogni settimana, suddivisi su almeno 3-5 giorni.

2. Sollevamento Pesi (Esercizi di Resistenza):

- Benefici: Gli esercizi di resistenza aiutano a sviluppare la massa muscolare, migliorare il metabolismo e promuovere la perdita di peso. Aumentano la forza muscolare e possono migliorare la composizione corporea, riducendo la percentuale di grasso corporeo.
- Consigliato: Includi almeno due giorni alla settimana di esercizi di resistenza che coinvolgano tutti i principali gruppi muscolari.

La combinazione di cardiofitness e sollevamento pesi offre un approccio completo per migliorare la salute del fegato grasso. Inoltre, è importante sottolineare che la perdita di peso, se necessaria, può essere un obiettivo importante nella gestione della steatosi epatica. L'esercizio aerobico contribuirà a bruciare calorie e a favorire la perdita di peso, mentre l'allenamento con i pesi può aiutare a mantenere la massa muscolare durante il processo di perdita di peso.

Prima di iniziare un nuovo programma di esercizio, è sempre consigliabile consultare il tuo medico o un professionista della salute, soprattutto se hai condizioni mediche preesistenti o stai seguendo trattamenti specifici per la steatosi epatica. Un professionista della salute può darti indicazioni personalizzate sulla base della tua situazione individuale e delle tue esigenze specifiche.

13. INPUT -> Quali farmaci devo assumere se ho la steatosi epatica?
OUTPUT:

Attualmente, non esiste una terapia farmacologica specifica approvata per il trattamento diretto della steatosi epatica non alcolica (NAFLD) o della steatosi epatica alcolica (ALD). Il trattamento principale per entrambe le forme di steatosi epatica si basa principalmente su modifiche dello stile di vita e sul controllo dei fattori di rischio associati.

Ecco alcune linee guida generali per il trattamento della steatosi epatica:

1. Modifiche dello Stile di Vita:

- Dieta Salutare: Adoptare una dieta equilibrata, limitando l'assunzione di zuccheri aggiunti, grassi saturi e alimenti ad alto contenuto calorico.
- Esercizio Fisico: Includere regolarmente l'attività fisica nella tua routine per migliorare la salute metabolica e favorire la perdita di peso, se necessario.
- Controllo del Peso: Mantenere un peso corporeo sano o cercare di perdere peso in caso di obesità o sovrappeso.
- Evitare l'Alcol: Ridurre o eliminare completamente il consumo di alcol in caso di steatosi epatica alcolica.

2. Controllo delle Condizioni Correlate:
- Gestire il diabete, se presente, attraverso una buona gestione della glicemia.
- Trattare l'ipertensione, se necessario, per mantenere la pressione arteriosa sotto controllo.
- Valutare e trattare altre condizioni mediche correlate, come l'iperlipidemia.

3. Monitoraggio Medico Regolare:
- Fornire un follow-up regolare con il tuo medico o un epatologo per monitorare la progressione della steatosi epatica e per valutare l'efficacia delle modifiche dello stile di vita.

4. Riduzione del Rischio di Malattie Cardiovascolari:
- Poiché la steatosi epatica è spesso associata a un aumento del rischio di malattie cardiovascolari, può essere necessario assumere farmaci come le statine o altri agenti ipolipemizzanti per gestire il rischio cardiaco.

5. Sperimentazione Clinica e Terapie Sperimentali: In alcune situazioni, il medico può consigliare la partecipazione a studi clinici o terapie sperimentali, poiché la ricerca continua a cercare trattamenti farmacologici specifici per la steatosi epatica.

È importante sottolineare che la gestione della steatosi epatica è un processo multidisciplinare che richiede la consulenza e il monitoraggio da parte di professionisti della salute, tra cui il tuo medico di famiglia o un epatologo. Non dovresti mai assumere farmaci per la steatosi epatica senza la supervisione e la prescrizione di un professionista medico, poiché non esiste una terapia farmacologica standard per questa condizione.

Il trattamento della steatosi epatica è altamente personalizzato e dovrebbe essere basato sulle tue condizioni mediche individuali, la gravità della steatosi epatica e altri fattori di rischio. Pertanto, è fondamentale lavorare a stretto contatto con il tuo medico per sviluppare un piano di trattamento adatto alle tue esigenze specifiche.

14. INPUT -> In che modo mi rendo conto che la mia steatosi epatica è in peggioramento? **OUTPUT:**

Per valutare se la tua steatosi epatica sta peggiorando, è importante monitorare attentamente la tua condizione e prestare attenzione a segni e sintomi che potrebbero indicare un cambiamento nella gravità della malattia. Ecco alcune cose da tenere d'occhio:

1. **Esami del Sangue**: I cambiamenti nei livelli ematici di enzimi epatici come l'ALT (alanina aminotransferasi) e l'AST (aspartato aminotransferasi) possono indicare un peggioramento della steatosi epatica. Un aumento costante o significativo di questi enzimi può essere un segno di infiammazione o danni al fegato.
2. **Esami di Imaging**: Gli esami di imaging come l'ecografia, la tomografia computerizzata (TC) o la risonanza magnetica (RM) possono essere utilizzati per valutare la gravità della steatosi epatica. Un aumento del grado di accumulo di grasso nel fegato o la presenza di cicatrici (fibrosi) possono indicare un peggioramento della malattia.
3. **Biopsia del Fegato**: In alcuni casi, il medico può raccomandare una biopsia del fegato per valutare la gravità della fibrosi e determinare se la steatosi epatica sta progredendo verso una forma più grave, come la cirrosi.
4. **Sintomi e Segni Fisici**: Puoi notare sintomi come affaticamento, dolore nella parte superiore destra dell'addome, ittero (ingiallimento della pelle e degli occhi), gonfiore addominale o prurito. Questi possono essere segnali di un peggioramento della steatosi epatica o di complicazioni associate.
5. **Modifiche dello Stile di Vita**: Presta attenzione a eventuali cambiamenti nelle tue abitudini di vita, come un aumento del consumo di alcol o un'eccessiva assunzione di cibi ad alto contenuto calorico e grassi saturi, che possono contribuire al peggioramento della steatosi epatica.
6. **Esami di Follow-up**: Se sei sotto la cura di un medico per la tua steatosi epatica, segui attentamente il piano di trattamento raccomandato e partecipa a visite di follow-up regolari. Durante queste visite, il medico può valutare la tua condizione e apportare modifiche al trattamento se necessario.

È importante sottolineare che la steatosi epatica è spesso asintomatica nelle fasi iniziali, il che significa che potresti non avvertire alcun sintomo. Pertanto, il monitoraggio regolare attraverso esami del sangue e esami di imaging è cruciale per rilevare e gestire tempestivamente il peggioramento della malattia.

Ricorda di discutere con il tuo medico o un epatologo quali indicatori e test di follow-up sono più appropriati per la tua situazione individuale e di seguire le raccomandazioni del professionista della salute per il monitoraggio e il trattamento della tua steatosi epatica.

15. INPUT -> In che modo mi rendo conto che la mia steatosi epatica è in miglioramento?
OUTPUT:

Per valutare se la tua steatosi epatica (fegato grasso) sta migliorando, dovresti prendere in considerazione diversi indicatori e segni positivi. Il miglioramento della steatosi epatica spesso implica una riduzione del grado di accumulo di grasso nel fegato e una diminuzione del rischio di complicazioni. Ecco alcuni modi per valutare se la tua condizione sta migliorando:

1. Esami del Sangue:
- Livelli degli Enzimi Epatici: Un miglioramento della steatosi epatica può essere riflessa da una normalizzazione o da una diminuzione dei livelli ematici di enzimi epatici come l'ALT (alanina aminotransferasi) e l'AST (aspartato aminotransferasi). Tuttavia, questi enzimi possono variare da persona a persona.

2. Esami di Imaging:
- Ecografia, TC o RM: Gli esami di imaging possono essere utilizzati per valutare la quantità di grasso nel fegato. Un miglioramento può essere indicato da una riduzione dell'accumulo di grasso.

3. Biopsia del Fegato:

- Fibrosi: Se hai subito una biopsia del fegato in precedenza, un miglioramento può essere evidente dalla diminuzione della fibrosi nel fegato. Tuttavia, la biopsia del fegato è solitamente riservata per casi più gravi.

4. Perdita di Peso:

- Controllo del Peso: Se hai perso peso in modo sano e mantenuto questa perdita di peso, ciò può essere un segno positivo di miglioramento della steatosi epatica, poiché la riduzione del peso corporeo è spesso associata a una riduzione del grasso nel fegato.

5. Modifiche dello Stile di Vita:

- Adesione alle Modifiche Dietetiche ed Esercizio Fisico: Il miglioramento della steatosi epatica è spesso correlato all'aderenza a modifiche dello stile di vita salutari, come una dieta equilibrata e l'esercizio fisico regolare.

6. Sintomi Ridotti o Assenza di Sintomi:

- Miglioramento dei Sintomi: Se avevi sintomi associati alla steatosi epatica, come affaticamento o malessere, potresti notare un miglioramento o la scomparsa di questi sintomi.

7. Monitoraggio Medico:

- Esami di Follow-up: Se sei in trattamento o sotto la supervisione di un medico, i tuoi esami di follow-up e le discussioni con il medico possono indicare se la tua steatosi epatica sta migliorando.

È importante sottolineare che il miglioramento della steatosi epatica può essere graduale e richiedere tempo. Le modifiche dello stile di vita, come una dieta sana e l'esercizio fisico, sono spesso fondamentali per il miglioramento. La gestione a lungo termine della steatosi epatica può essere necessaria per mantenere i benefici ottenuti.

Ricorda sempre di consultare il tuo medico o un epatologo per una valutazione accurata e per stabilire se la tua steatosi epatica sta migliorando. La loro supervisione e il monitoraggio regolare sono essenziali per garantire un trattamento adeguato e la gestione della tua condizione.

Supplementary material: QUESTIONS POSED TO CHATGPT (ENGLISH TRANSLATION)

1. **INPUT** -> I have been diagnosed with steatotic liver disease. Should I be referred to a liver specialist? Should I have additional blood tests or radiological examinations?

OUTPUT:

I am happy to give you some information about hepatic steatosis, but remember that I am not a doctor and my answers are for informational purposes only. It is important to consult a physician or hepatologist for specific advice and treatment based on your individual situation.

Hepatic steatosis, commonly known as "fatty liver," is a condition in which fat accumulates in liver cells. There are two main types of hepatic steatosis:

1. Non-alcoholic liver steatosis (NAFLD): This is the most common form of liver steatosis and is not caused by alcohol abuse. It can be associated with factors such as obesity, diabetes, hypertension, and hyperlipidemia.
2. Alcoholic liver steatosis (ALD): This form is caused by chronic alcohol abuse.

The management of hepatic steatosis will depend on its cause and the severity of the condition. However, in general, here are some common measures that can be recommended:

1. Lifestyle changes: Weight loss, regular exercise and a balanced diet can help reduce fat in the liver. These measures may be especially important if you have NAFLD.
2. Limiting alcohol intake: If you have ALD, it will be important to reduce or eliminate alcohol intake.
3. Medical monitoring: It will be important to have regular doctor visits to monitor your condition and assess your progress.
4. Blood tests and imaging: Your doctor may recommend that you have blood tests to monitor your liver function and may require imaging tests such as ultrasound, computed tomography (CT) or magnetic resonance imaging (MRI) to assess the degree of fat accumulation in the liver.
5. Consultation with a hepatologist: If your condition is more serious or complicated, you may need to consult a hepatologist, a doctor who specializes in liver disease. The hepatologist will be able to give you more in-depth advice and plan specific treatment if necessary.

Remember that the management of hepatic steatosis can vary from person to person, so it is important to follow your doctor's instructions carefully to achieve the best possible outcome. Do not hesitate to contact your medical professional to discuss your situation and treatment options further

2. **INPUT ->** How much weight should I lose if I have steatotic liver disease?

OUTPUT:

The amount of weight you should lose if you have liver steatosis will depend on your individual situation, the severity of the condition, and other factors, such as your current body weight, age, sex, and general health condition. There is no universal answer in terms of how many pounds you should lose.

In general, weight loss can be helpful in the management of nonalcoholic hepatic steatosis (NAFLD), especially if you are overweight or obese. Even a modest reduction in body weight can lead to significant

improvements in liver health. However, it is important to emphasize that healthy, sustainable weight loss should be gradually achieved and should be supervised by a medical professional or registered dietitian.

The amount of weight you should aim to lose may vary, but in many cases, a loss of 5-10% of total body weight can have a positive impact on liver health. For example, if your current body weight is 90 kg, a 5-10% weight loss would be equivalent to 4.5-9 kg. However, it is important to discuss with your physician or a qualified health professional to establish specific weight loss goals based on your situation and needs.

The key to effective and lasting weight loss is to adopt a healthy lifestyle that includes a balanced diet, regular exercise, and sustainable changes in eating habits. The goal should be to improve overall health, not just liver condition. A physician or dietitian can help you develop a personalized plan that takes into account your specific needs and goals.

3. **INPUT ->** Which diet should I follow if I have steatotic liver disease?

OUPUT:

The diet for hepatic steatosis should be developed in collaboration with a medical professional or registered dietitian, as the exact diet may vary depending on the severity of your condition, your individual needs, and other health factors. However, I can give you some general guidelines for a diet that can help manage hepatic steatosis:

- Reduce your caloric intake: If you are overweight or obese, weight loss may be an important goal. Gradually reducing your caloric apportion can help you lose weight in a healthy and sustainable way. Consult a health professional to determine your optimal daily caloric intake.
- Limit your intake of saturated and trans fats: Reduce your intake of foods high in saturated and trans fats, such as fatty meats, whole dairy products, fried foods, and baked goods with hydrogenated fats. Instead, opt for healthier fats such as those found in avocados, nuts, seeds and vegetable oils such as olive oil.
- Choose lean protein: Lean proteins, such as skinless chicken, fish, tofu, and legumes, may be a better choice than fatty red meats. Reduce your intake of red and processed meat.
- Increase fiber intake: Fiber can help control weight and improve liver health. Choose fiber-rich foods such as fruits, vegetables, whole grains, legumes and nuts.
- Limit intake of added sugars: Reduce your intake of added sugars, such as those found in sugary drinks, sweets, cookies and processed foods.
- Monitor carbohydrate intake: Some people with hepatic steatosis may benefit from moderately limiting carbohydrate intake, especially those with a high glycemic index. A health professional can help you plan a carbohydrate intake that is appropriate for your situation.
- Avoid alcohol: If you have alcoholic liver steatosis (ALD), it is essential to eliminate or drastically reduce alcohol consumption.
- Drink water: Keep hydrated by drinking plenty of water throughout the day.
- Break up meals: Eating small, frequent meals can help maintain stable blood sugar levels and feelings of fullness.

Remember that these are just general tips and that the diet should be tailored to your specific needs. It is important to consult with a medical
professional or registered dietitian who can assess your individual situation and develop a tailor-made diet plan for you. In addition, regular
monitoring of your condition by a physician is essential to ensure that you are getting the results you want and to make any adjustments to your
dietary regimen

4. **INPUT** -> Which foods should I avoid if I have steatotic liver disease?

OUTPUT:

If you have liver steatosis, you should avoid or limit your intake of certain foods and drinks that can worsen your fatty liver condition. Here are some foods and beverages you should consider avoiding or consuming very moderately:

- Alcohol: If you have alcoholic liver steatosis (ALD), it is critical to avoid alcohol completely. Alcohol can further damage your liver and make the condition worse.
- Foods high in saturated fats: Reduce or eliminate your consumption of foods high in saturated fats, such as fatty meats, butter, full-fat cheeses, and baked goods containing hydrogenated saturated fats.
- Added sugars: Limit your intake of added sugars, such as those found in sugary drinks, cakes, cookies, sugary breakfast cereals and other sweet products.
- Fried foods: Avoid foods fried or cooked in saturated fats or oils high in saturated fats.

- Highly processed foods: Reduce your intake of highly processed foods, such as packaged snacks, fast foods, and frozen convenience foods, as they often contain saturated fats, added sugars, and additives that may not be beneficial to the liver.

- Red and processed meat: Limit your consumption of fatty red meat and processed meats such as sausages, bacon and hot dogs.

- Energy drinks: Avoid energy drinks and sugary carbonated drinks, as they are often high in added sugar and may contribute to overweight.

- Salts and foods high in sodium: Reduce your consumption of salt and foods high in sodium, as high sodium consumption can lead to health problems, including hypertension.

- Foods with a high glycemic index: Some studies suggest that a diet with a high glycemic index may adversely affect hepatic steatosis. Try to limit foods with a high glycemic index, such as white bread, white rice, and refined grains.

- Alcohol-free alcoholic drinks: Some alcohol-free drinks may contain added sugars or artificial sweeteners that may not be beneficial. Read ingredient labels.

In general, you should eat a balanced diet rich in fruits, vegetables, whole grains, lean protein and healthy fats such as those present in avocados, nuts and vegetable oils. A dietitian or health professional can help you develop an eating plan specific to your individual needs and management of hepatic steatosis.

5. **INPUT** -> Can I drink alcohol if I have steatotic liver disease?

OUTPUT:

If you have been diagnosed with hepatic steatosis, it is critical to make informed decisions about your diet and alcohol consumption. In general, alcohol should be avoided or consumed very moderately if you have hepatic steatosis, regardless of its form (alcoholic or nonalcoholic). Here are some important considerations:

- Alcoholic Hepatic Steatosis (ALD): If you have the alcoholic form of hepatic steatosis, it is extremely advisable to avoid alcohol completely. Alcohol intake can worsen the condition of your liver and contribute to the development of more serious liver disease such as alcoholic hepatitis and cirrhosis. Total alcohol withdrawal is the primary treatment for ALD.
- Non-Alcoholic Hepatic Steatosis (NAFLD): Although the nonalcoholic form of hepatic steatosis (NAFLD) is not caused by alcohol abuse, it is still important to limit or eliminate alcohol consumption. Alcohol can put additional stress on your liver and can make the management of NAFLD more difficult. In addition, NAFLD is often associated with risk factors such as obesity, diabetes, and metabolic syndrome, and alcohol can negatively affect these factors.
- Medical Consultation: Your decision to consume alcohol should be discussed in detail with a medical professional, such as a hepatologist or gastroenterologist. The doctor can assess the severity of your condition, risk factors and give you personalized advice based on your specific situation. In many cases, doctors recommend avoiding alcohol completely to protect the liver.
- Constant Monitoring: Regardless of your decision about alcohol, it is important to have regular medical monitoring visits to assess your liver health and make any treatment or lifestyle changes based on your response to treatment.

In summary, avoiding alcohol completely is often the safest choice to protect your liver health if you have hepatic steatosis. However, the final decision should be made in consultation with your physician, who will consider all factors relevant to your clinical situation.

6. **INPUT** -> Which foods have a positive effect on steatotic liver disease?

OUTPUT:

A balanced and targeted diet can have a positive effect on hepatic steatosis, helping to improve liver health. Here are some foods that may be beneficial for liver steatosis:

- Fruits and vegetables: They are rich in antioxidants, vitamins, and fiber, which can help reduce inflammation and improve liver health. Choose a variety of colorful fruits and vegetables to maximize the benefits.

- Whole grains: Foods such as whole grain bread, brown rice, oats, and quinoa are sources of complex carbohydrates and fiber that can help control blood sugar levels and promote weight loss.

- Lean protein: Lean meat, fish, skinless poultry, tofu, and legumes are good sources of lean protein that can be included in a diet for hepatic steatosis.

- Nuts and seeds: They are rich in healthy fats, including omega-3 fatty acids, which can have a positive effect on liver health. Nuts are also caloric, so it is important to consume them in moderation.

- Healthy vegetable oils: Extra virgin olive oil and flaxseed oil are examples of healthy vegetable oils that can be used for seasoning foods and cooking.

- Fatty fish: Fatty fish such as salmon, tuna, and sardines are good sources of omega-3 fatty acids, which may have anti-inflammatory proprieties and contribute to liver health.

- Green tea: Green tea is rich in antioxidants and may help reduce inflammation in the liver.

- Low-fat milk and dairy products: Skim milk, low-fat yogurt and low-fat cheese may be better choices than their high-fat equivalents.

- Fiber-rich foods: Fiber-rich foods, such as legumes, oats, and green leafy vegetables, can help regulate intestinal transit and improve fat metabolism.

- Apple cider vinegar: Some studies suggest that apple cider vinegar may have positive effects on weight management and liver fat reduction.

Remember that the key to an effective diet for liver steatosis is moderation and variety. In addition, it is important to consult a medical professional or registered dietitian to develop an individualized eating plan based on your individual situation and health goals.

7. **INPUT ->** Can I use herbal remedies if I have steatotic liver disease?

OUTPUT:

The use of herbal remedies for hepatic steatosis (fatty liver) should be done with caution and under the supervision of a medical professional. While some herbs may have benefits for liver health, it is important to consider several factors:

1. Medical Consultation: Before starting any herbal treatment for hepatic steatosis, it is critical to consult your doctor or a hepatologist. This is important because you need to confirm the diagnosis and determine the underlying cause of your liver steatosis. In addition, your doctor can evaluate potential interactions between herbal supplements and other medications you may be taking for other medical conditions.
2. Quality and Safety: Be sure to use high-quality herbal products from reputable sources. The safety and purity of herbal products can vary widely, so it is important to shop from trusted manufacturers or retailers.
3. Drug Interactions: Some herbal remedies may interact with medications or other medical treatments you are taking. Inform your doctor or health care professional about the herbal remedies you plan to use to avoid potentially dangerous interactions.
4. Side Effects: Herbal supplements may also have side effects or allergic reactions. Closely monitor your response to herbal remedies and discontinue use if you notice side effects.
5. Limited Scientific Evidence: Scientific research on the specific effects of herbal supplements on liver steatosis is often limited or inconclusive. Many herbal remedies have not been subjected to rigorous clinical trials to evaluate their efficacy.
6. Integrated Approach: The use of herbal remedies should be an integral part of a comprehensive liver steatosis management plan, which may also include lifestyle modifications such as a balanced diet, exercise, and weight management.

Some herbs commonly associated with liver health include milk thistle, dandelion, turmeric, and red clover. However, it is important to emphasize that treatment of hepatic steatosis should be based on sound clinical evidence and supervised by a medical professional.

In summary, if you wish to use herbal remedies for hepatic steatosis, always discuss it with your physician or a hepatologist and follow their recommendations. Medical supervision is essential to ensure that the treatment is safe and effective and is appropriate for your individual situation.

8. **INPUT ->** Can I drink coffee if I have steatotic liver disease?

OUTPUT:

Moderate coffee consumption is generally considered safe for people with hepatic steatosis (fatty liver), whether it is nonalcoholic liver steatosis (NAFLD) or alcoholic liver steatosis (ALD). In fact, some research suggests that coffee may have benefits for liver health. However, it is important to make some observations:

1. Moderation: As with many things in the diet, moderation is the key. Consuming coffee in excessive amounts may not be healthy for the liver or overall health. Usually, one or two cups of coffee a day is considered a moderate amount.
2. Individual Tolerance: Tolerance to coffee can vary from person to person. Some people may be more sensitive to the stimulant effects of caffeine, so it is important to listen to your body and adjust your coffee consumption according to your reactions.
3. Potential Benefits: Some studies have suggested that coffee may have beneficial effects on liver health. In particular, it has been noted that coffee may help reduce the risk of developing NAFLD and may have a positive impact on disease progression. However, the effects may vary from person to person.
4. Beware of Additives: When drinking coffee, it is important to consider additives such as sugar and creamer. Adding large amounts of sugar and saturated fat can have negative effects on overall health, including liver health.
5. Medical Consultation: If you have specific concerns about your liver health or if you are undergoing medical treatment for hepatic steatosis, it is always a good idea to consult your doctor or a hepatologist for personalized guidance on coffee consumption.

In summary, coffee can be consumed in moderation as part of a balanced diet, and it may even have benefits for fatty liver health. However, it is important to be aware of your limitations and to consult a

health professional if you have specific questions or concerns about the relationship between coffee and hepatic steatosis.

9. **INPUT ->** Can I smoke if I have steatotic liver disease?
OUTPUT:

Smoking is detrimental to overall health and can have adverse effects on the liver, including fatty liver (hepatic steatosis). Here are some important considerations:

1. Negative Effects of Smoking on the Liver: Cigarette smoking is associated with a number of negative effects on the liver, including an increased risk of liver disease, including progression of hepatic steatosis to more severe forms such as alcoholic hepatitis or cirrhosis. Smoking can also contribute to chronic inflammation in the liver.
2. Interactions with Other Conditions: If you have hepatic steatosis, it is likely that you also have other risk factors such as obesity, diabetes, or hypertension. Smoking can make these conditions worse, making it more difficult to control liver steatosis and increasing the risk of complications.
3. Effects on Fat Metabolism: Cigarette smoking can alter fat metabolism in the liver, contributing to fat storage. This is particularly relevant in cases of hepatic steatosis, where fat management in the liver is already impaired.
4. Quit Smoking: The best choice for your overall health and management of hepatic steatosis would be to quit smoking completely. Quitting smoking can improve your liver health and reduce the risk of serious liver complications in the long term.
5. Stop Smoking Support: If you wish to quit smoking, seek support from a medical professional or tobacco cessation program. There are many resources available to help you quit smoking effectively.

In summary, if you have liver steatosis, quitting smoking is an important choice to improve the health of your liver and your body as a whole. Consult your doctor for support in your decision to quit smoking and for specific advice on managing your hepatic steatosis.

10. **INPUT ->** How much exercise should I do if I have steatotic liver disease?
OUTPUT:

Physical activity is an important component in the management of hepatic steatosis (fatty liver), particularly if it is nonalcoholic hepatic steatosis (NAFLD). Exercise can help improve overall health, reduce body weight, improve insulin sensitivity, and promote liver health. However, the type and amount of physical activity may vary depending on your individual condition and fitness level.

Here are some general guidelines on the amount and type of physical activity to consider:

1. Medical Advice: Before starting any exercise program, it is important to consult your doctor or a health professional to make sure it is safe for you, especially if you have pre-existing medical conditions or are undergoing specific treatments for hepatic steatosis.
2. Aerobic Activity: Aerobic exercises such as walking, swimming, jogging, cycling or dancing can be very beneficial for improving liver health and weight loss. You should aim for 150-300 minutes of moderate aerobic activity or 75-150 minutes of vigorous activity each week, spread out over at least 3-5 days.
3. Strength Exercises: Strength or resistance exercises, such as lifting weights or using resistance machines, can help build muscle mass and improve metabolism. It is recommended to include at least two days a week of strength exercises involving all major muscle groups.
4. Flexibility and Balance: Stretching exercises and activities such as yoga or tai chi can help improve flexibility, balance, and coordination.
5. Graduality: If you are inactive or have limited fitness, start gradually and increase the intensity and duration of physical activity as you feel more comfortable. The goal is to create an exercise program that is sustainable over time.
6. Variety: Choose a variety of activities that you enjoy to make exercise more enjoyable and sustainable over the long term.
7. Persistence: Consistency is key. Try to maintain a regular exercise program over time.
8. Hydration: Make sure you stay well hydrated during and after exercise.
9. Periodic Medical Checkup: Continue to monitor your condition with your doctor and seek his or her advice on how to adjust your exercise program based on your response and health goals.

In general, exercise is an important component of improving fatty liver health, but it is essential to be aware of your limitations and to consult with a health professional to develop a personalized exercise plan.

11. **INPUT ->** Which type of exercise is better for steatotic liver disease?

OUTPUT:

The best type of physical activity for hepatic steatosis (fatty liver) is a combination of aerobic (cardio) exercises, resistance (strength) exercises, and activities that improve flexibility and balance. A comprehensive physical activity program can provide several benefits to liver health and the management of hepatic steatosis. Here is how each type of activity can be beneficial:

1. Aerobic (Cardio) Exercises:

- Walking: Walking is an affordable option and can be suitable for almost anyone.
- Swimming: This is a low-impact form of exercise that involves many muscles.
- Cycling: Good for the heart and for burning calories.
- Running: Can be more intense but effective for improving cardiovascular health.
- Dancing: A fun mode of aerobic exercise.

2. Resistance (Strength) Exercises:

- Weight Lifting: You can use free weights or resistance machines.
- Body Weight Exercises: Such as push-ups, squats and lunges.
- Yoga or Pilates: These can include strength exercises for the core and general body.

3. Exercises for Flexibility and Balance:

- Yoga: Promotes flexibility and relaxation.
- Tai Chi: Helps improve balance and coordination.
- Stretching: Helps maintain muscle flexibility and prevent injury.

The goal is to develop a physical activity program that combines these different modalities to achieve a full range of health benefits. Here are some general guidelines:

- Frequency: Try to exercise at least 3-5 days a week.
- Duration: The goal is at least 150-300 minutes of moderate aerobic exercise or 75-150 minutes of intense exercise each week, spread out over at least 3-5 days. Also include at least two days per week of resistance exercise involving all major muscle groups.
- Graduality: If you are inactive or have limited fitness, start gradually and increase intensity and duration as you feel more comfortable.
- Medical Consultation: Consult your doctor or a health professional before starting a new exercise program, especially if you have pre-existing medical conditions or are undergoing specific treatments for hepatic steatosis.

In summary, a combination of aerobic exercises, resistance exercises, and activities for flexibility and balance is the best approach to improving liver health and managing liver steatosis. A health professional can help you develop an exercise plan tailored to your individual needs and situation.

12. **INPUT ->** Should I do cardio or lift weights for steatotic liver disease?

OUTPUT:

If you have hepatic steatosis (fatty liver), it is advisable to combine both cardiofitness (aerobic) and weight training (resistance) in your exercise program. Both types of physical activity can be beneficial for the management of hepatic steatosis, but they play slightly different roles. That's why it's helpful to include both:

1. Cardiofitness (Aerobic Exercises):

- Benefits: Aerobic exercises, such as walking, swimming, cycling or running, help burn calories, improve cardiovascular capacity, reduce body weight and increase insulin sensitivity. They can also help reduce fat accumulation in the liver.
- Recommended: Try to commit to at least 150-300 minutes of moderate aerobic exercise or 75-150 minutes of intense exercise each week, spread out over at least 3-5 days.

2. Weight Lifting (Resistance Exercises):

- Benefits: Resistance exercises help build muscle mass, improve metabolism and promote weight loss. They increase muscle strength and can improve body composition, reducing body fat percentage.
- Recommended: Include at least two days a week of resistance exercise involving all major muscle groups.

The combination of cardio and weight lifting offers a comprehensive approach to improving fatty liver health. In addition, it is important to note that weight loss, if needed, can be an important goal in the management of hepatic steatosis. Aerobic exercise will help burn calories and promote weight loss, while weight training can help maintain muscle mass during the weight loss process.

Before starting a new exercise program, it is always advisable to consult your doctor or a health professional, especially if you have pre-existing medical conditions or are undergoing specific treatments for hepatic steatosis. A health professional can give you personalized guidance based on your individual situation and specific needs.

13. **INPUT ->** Which drugs should I take if I have steatotic liver disease?
OUTPUT:

Currently, there is no specific drug therapy approved for the direct treatment of nonalcoholic hepatic steatosis (NAFLD) or alcoholic liver steatosis (ALD). The main treatment for both forms of hepatic steatosis relies mainly on lifestyle modifications and control of associated risk factors.

Here are some general guidelines for the treatment of hepatic steatosis:

1. Lifestyle Modifications:

- Healthy Diet: Adopt a balanced diet, limiting intake of added sugars, saturated fats and high-calorie foods.
- Exercise: Include regular physical activity in your routine to improve metabolic health and promote weight loss, if necessary.
- Weight Control: Maintain a healthy body weight or try to lose weight if you are obese or overweight.
- Avoiding Alcohol: Reduce or completely eliminate alcohol consumption if you have alcoholic liver steatosis.

2. Control of Related Conditions:

- Manage diabetes, if present, through good blood glucose management.
- Treat hypertension, if necessary, to keep blood pressure under control.
- Assess and treat other related medical conditions, such as hyperlipidemia.

3. Regular Medical Monitoring:

- Provide regular follow-up with your physician or a hepatologist to monitor the progression of liver steatosis and to assess the effectiveness of lifestyle modifications.

4. Reducing the Risk of Cardiovascular Disease:

- Because liver steatosis is often associated with an increased risk of cardiovascular disease, you may need to take medications such as statins or other hypolipidemic agents to manage your heart risk.

5. Clinical Trials and Experimental Therapies: In some situations, the physician may recommend participation in clinical trials or experimental therapies as research continues to seek specific drug treatments for hepatic steatosis.

Importantly, the management of hepatic steatosis is a multidisciplinary process that requires consultation and monitoring by health professionals, including your family physician or a hepatologist. You should never take medications for hepatic steatosis without the supervision and prescription of a medical professional, as there is no standard drug therapy for this condition.

Treatment of hepatic steatosis is highly individualized and should be based on your individual medical condition, the severity of hepatic steatosis, and other risk factors. Therefore, it is critical to work closely with your physician to develop a treatment plan tailored to your specific needs.

14. **INPUT ->** How do I understand if steatotic liver disease is worsening?
OUTPUT:

To assess whether your liver steatosis is worsening, it is important to monitor your condition closely and pay attention to signs and symptoms that might indicate a change in the severity of the disease. Here are some things to watch for:

1. Blood Tests: Changes in blood levels of liver enzymes such as ALT (alanine aminotransferase) and AST (aspartate aminotransferase) may indicate worsening liver steatosis. A steady or significant increase in these enzymes may be a sign of inflammation or liver damage.
2. Imaging Examinations: Imaging examinations such as ultrasound, computed tomography (CT) or magnetic resonance imaging (MRI) can be used to assess the severity of hepatic steatosis. An increase in the degree of fat accumulation in the liver or the presence of scarring (fibrosis) may indicate worsening of the disease.
3. Liver Biopsy: In some cases, the physician may recommend a liver biopsy to assess the severity of fibrosis and determine if the liver steatosis is progressing to a more severe form, such as cirrhosis.
4. Symptoms and Physical Signs: You may notice symptoms such as fatigue, pain in the right upper abdomen, jaundice (yellowing of the skin and eyes), abdominal swelling, or itching. These may be signs of worsening hepatic steatosis or associated complications.
5. Lifestyle Changes: Pay attention to any changes in your lifestyle habits, such as increased alcohol consumption or excessive intake of high-calorie foods and saturated fats, which can contribute to worsening hepatic steatosis.
6. Follow-up Examinations: If you are under a doctor's care for your liver steatosis, follow the recommended treatment plan carefully and attend regular follow-up visits. During these visits, your doctor can evaluate your condition and make treatment changes if necessary.

It is important to note that hepatic steatosis is often asymptomatic in the early stages, which means you may not experience any symptoms. Therefore, regular monitoring through blood tests and imaging examinations is crucial to detect and manage worsening disease early.

Remember to discuss with your doctor or a hepatologist which indicators and follow-up tests are most appropriate for your individual situation and to follow the health professional's recommendations for monitoring and treating your hepatic steatosis.

15. **INPUT ->** How do I understand if steatotic liver disease is improving?
OUTPUT:

To assess whether your hepatic steatosis (fatty liver) is improving, you should consider several positive indicators and signs. Improvement in hepatic steatosis often implies a reduction in the degree of fat accumulation in the liver and a decreased risk of complications. Here are some ways to assess whether your condition is improving:

1. Blood tests:

- Liver Enzyme Levels: An improvement in liver steatosis may be reflected by a normalization or decrease in blood levels of liver enzymes such as ALT (alanine aminotransferase) and AST (aspartate aminotransferase). However, these enzymes may vary from person to person.

2. Imaging examinations:

- Ultrasound, CT or MRI: Imaging examinations can be used to assess the amount of fat in the liver. An improvement may be indicated by a reduction in fat accumulation.

3. Liver Biopsy:

- Fibrosis: If you have had a liver biopsy previously, an improvement may be evident by a decrease in fibrosis in the liver. However, liver biopsy is usually reserved for more severe cases.

4. Weight Loss:

- Weight Control: If you have lost weight in a healthy way and maintained this weight loss, this may be a positive sign of improvement in hepatic steatosis, as reduction in body weight is often associated with a reduction in fat in the liver.

5. Lifestyle Modifications:

- Adherence to Dietary Modifications and Exercise: Improvement in hepatic steatosis is often related to adherence to healthy lifestyle modifications, such as a balanced diet and regular exercise.

6. Reduced Symptoms or Absence of Symptoms:

- Improved Symptoms: If you had symptoms associated with liver steatosis, such as fatigue or malaise, you may notice an improvement or disappearance of these symptoms.

7. Medical Monitoring:

- Follow-up Exams: If you are being treated or supervised by a doctor, your follow-up exams and discussions with your doctor may indicate whether your liver steatosis is improving.

It is important to note that improvement in liver steatosis can be gradual and take time. Lifestyle modifications, such as a healthy diet and exercise, are often key to improvement. Long-term management of hepatic steatosis may be necessary to maintain the benefits achieved.

Always remember to see your doctor or a hepatologist for a thorough evaluation and to determine if your liver steatosis is improving. Their supervision and regular monitoring are essential to ensure proper treatment and management of your condition.
